# Molecular Interaction and Biological Activity of Fatty Acids and Sterols: An *In Silico* and *In Vitro* Approach Against *Haemonchus contortus*

**DOI:** 10.3390/ph19010140

**Published:** 2026-01-14

**Authors:** Susan Yaracet Páez-León, Alexandre Cardoso-Taketa, Abraham Madariaga-Mazón, Adriana Morales-Martínez, Juan Felipe de Jesús Torres-Acosta, Gabriela Mancilla-Montelongo, Víctor Manuel Hernández-Velázquez, Gabriel Navarrete-Vázquez, Elba Villegas, Liliana Aguilar-Marcelino

**Affiliations:** 1 Centro de Investigación en Biotecnología, Universidad Autónoma del Estado de Morelos, Av. Universidad 1001, Cuernavaca 62209, Morelos, Mexico; susan.paezleo@fcbiologicas.uaem.edu.mx (S.Y.P.-L.); ataketa@uaem.mx (A.C.-T.); adriana.morales@uaem.mx (A.M.-M.); vmanuelh@uaem.mx (V.M.H.-V.); elbav@uaem.mx (E.V.); 2 Unidad Mérida del Instituto de Química, Universidad Nacional Autónoma de Mexico, Km. 5.5 Carr. Sierra Papacal-Chuburná Pto., Sierra Papacal 97302, Yucatán, Mexico; amadariaga@iquimica.unam.mx; 3 Facultad de Medicina Veterinaria y Zootecnia, Universidad Autónoma de Yucatán, Carretera Mérida-Xmatkuil Km. 15.5. Xmatkuil, Mérida 97315, Yucatán, Mexico; tacosta@correo.uady.mx; 4 SECIHTI, Facultad de Medicina Veterinaria y Zootecnia, Universidad Autónoma de Yucatán, Carretera Mérida-Xmatkuil Km. 15.5. Xmatkuil, Mérida 97315, Yucatán, Mexico; maria.mancilla@correo.uady.mx; 5 Facultad de Farmacia, Universidad Autónoma del Estado de Morelos, Av. Universidad 1001, Cuernavaca 62209, Morelos, Mexico; gabriel_navarrete@uaem.mx; 6 Centro Nacional de Investigación Disciplinaria en Salud Animal e Inocuidad, Sede Palo Alto Instituto Nacional de Investigaciones Forestales Agrícolas y Pecuarias (INIFAP), Km 15.5 Carretera Federal México-Toluca, Cuajimalpa de Morelos, Mexico City 05110, Mexico

**Keywords:** nematodes, alternative control, synthetic compounds, molecular docking

## Abstract

**Background:** *Haemonchus contortus* is a gastrointestinal nematode that affects small ruminants and exhibits widespread resistance to commercial anthelmintics. This has driven interest in natural compounds such as fatty acids and sterols; however, their biological relevance against resistant parasite strains remains insufficiently understood. **Methods:** The nematicidal potential of four fatty acids (palmitic, linoleic, pentadecanoic, and stearic acids) and two sterols (β-sitosterol and ergosterol), all of them commercially available in Mexico, was evaluated against infective L3 larvae of a benzimidazole-resistant *H. contortus* strain. *In vitro* larval mortality and migration inhibition assays were performed, and molecular docking analyses were conducted to explore interactions with the glutamate-gated chloride channel (GluCl) using AutoDock4. Statistical analyses were performed using ANOVA followed by Tukey’s post hoc test (*p* < 0.05). **Results:** Molecular docking indicated strong binding affinities of ergosterol and β-sitosterol to GluCl, comparable to that of ivermectin. *In vitro* assays showed that fatty acids, particularly linoleic acid, produced more pronounced effects on larval motility, suggesting predominantly nematostatic activity. No clear dose–response relationship was observed in migration assays, and *in vitro* mortality remained limited across treatments. **Conclusions:** The results highlight a disconnect between *in silico* binding affinity and *in vitro* biological activity, particularly in a drug-resistant *H. contortus* strain. Integrating *in vitro* bioassays with computational approaches provides valuable mechanistic insight but also underscores the limitations of affinity-based predictions for assessing anthelmintic efficacy.

## 1. Introduction

Haemonchosis is a disease that contributes to the development of anemia in small ruminants. It is an infection caused by the nematode *Haemonchus contortus*, which adapts to a wide range of climatic zones but is most prevalent in tropical environments [1]. Recent studies indicate that climate change is allowing *H. contortus* to survive and thrive in areas that were previously characterized as low-risk areas [2]. Current strategies for controlling haemonchosis are based primarily on the use of synthetic anthelmintic drugs developed during the second half of the 20th century.

Presently, there are three main classes of anthelmintics that are used for the treatment of this disease in small ruminants, these being the benzimidazoles (BZs), macrocyclic lactones (MLs), and cholinergic agonists (especially levamisole: LEV). These drugs target diverse proteins, including β-tubulin, acetylcholine receptors, estrogenic receptors, and receptors involved in synaptic neurotransmission, called Cys-loop ligand-gated, which block chloride channels (LGCCs) [3,4].

The extensive and uncontrolled use of these drugs has led to increased resistance in several parasitic nematodes, which has become an emerging concern worldwide [5]. Resistance can be considered a change in the response to drug treatment, such that efficacy is reduced compared to that observed when the drug was first introduced in the field.

*H. contortus* is the most studied trichostrongylid nematode to address resistance to anthelmintics. This is partly because this species has a high capacity to develop resistance to all major classes of drugs and can be used as a model to ascertain the efficiency of alternative treatment methods [6,7].

Among promising alternative substances that have shown to have the potential to act as anthelmintics, due to their bioactive properties, are certain fatty acids and sterols. Fatty acids (FAs) are ubiquitous in nature and play a crucial role in vital processes. They are a basic component of lipids and most of them contain an even number of carbon atoms, ranging from 4 to 28 [8]. In addition, several types of FAs and their derivatives have been reported to have nematicidal activity [8,9,10].

On the other hand, sterols are substances found naturally in the animal and plant worlds, as well as in fungi and yeasts. The structural characteristic of sterols is the steroid nucleus formed by four fused rings, three with six carbon atoms and one with five, which carries a hydroxyl group at C-3 and most of the cholestane skeleton (C_27_H_48_) [11]. Sterols are components of cell membranes and serve as precursors for a variety of products with specific biological activities [12]. β-sitosterol has been reported to exhibit anthelmintic activity against various nematode species [9,13,14].

In addition to evaluating the biological activity of these compounds *in vitro*, it is important to understand how these compounds bind to small molecules and specific receptors to potentially enhance their effectiveness. This can be achieved using molecular docking analysis and basic tools such as a search algorithm and an energy scoring function for generating and evaluating ligand pose.

The objectives of the present study were to evaluate the anthelmintic properties of four commonly found FAs and two sterols using *in vitro* systems, and to perform an *in silico* analysis of these compounds as a basis for the mechanism of action at the ivermectin binding site of the glutamate-dependent chloride (GluCl) homology model of *H. contortus*. We selected the GluCl channels as these are an important target for anthelmintics derived from macrocyclic lactones.

## 2. Results

### 2.1. ADMET Analysis

The physicochemical and drug-likeness properties of six compounds, Palmitic acid (APA), Pentadecanoic acid (APE), Stearic acid (AES), Linoleic acid (ALN), β-sitosterol (BST), and Ergosterol (ERG), were evaluated using the online SwissADME tool. The results are summarized in Table 1. The compounds had molecular weights between 242.40 and 414.71 g/mol, all below the critical threshold of 500 g/mol established by Lipinski’s rule. However, a high number of rotatable bonds were observed in the fatty acids (APA, APE, AES, and ALN), with values above 13, which may negatively influence oral bioavailability due to greater molecular flexibility. In contrast, the sterols BST and ERG showed fewer rotatable bonds (6 and 4, respectively), which may favor their conformational stability. Regarding the ability to form hydrogen bonds, all compounds had ≤2 acceptors and a single donor, within the acceptable range for good intestinal permeability and absorption. The bioavailability score was 0.85 for fatty acids, while BST and ERG had a lower value (0.55), suggesting potentially more limited oral absorption for the latter. No PAINS alerts were detected in any of the compounds, which is favorable as it reduces the likelihood of false positives in biological assays. However, both BST and ERG presented a structural alert according to the Brenk filter, indicating the presence of possible toxic or undesirable substructures. Finally, synthetic accessibility was favorable for fatty acids (values between 2.20 and 2.54), suggesting that these compounds would be relatively easy to obtain by chemical synthesis. In contrast, sterols showed greater synthetic complexity, with values above 6.3, representing a possible limitation in their development as therapeutic candidates.

### 2.2. Molecular Docking

The binding energy values obtained for the compounds evaluated against the target protein GluCl channel from *H. contortus* are shown in Table 2. In general, sterols were found to have the highest affinities, while fatty acids showed weaker interactions with both molecular targets. Ivermectin showed the highest binding affinity (−13.57 kcal/mol), followed by ergosterol (−10.25 kcal/mol) and β-sitosterol (−10.18 kcal/mol). Fatty acids showed affinities, with values ranging from −4.36 to −4.59 kcal/mol.

**Figure 1:**
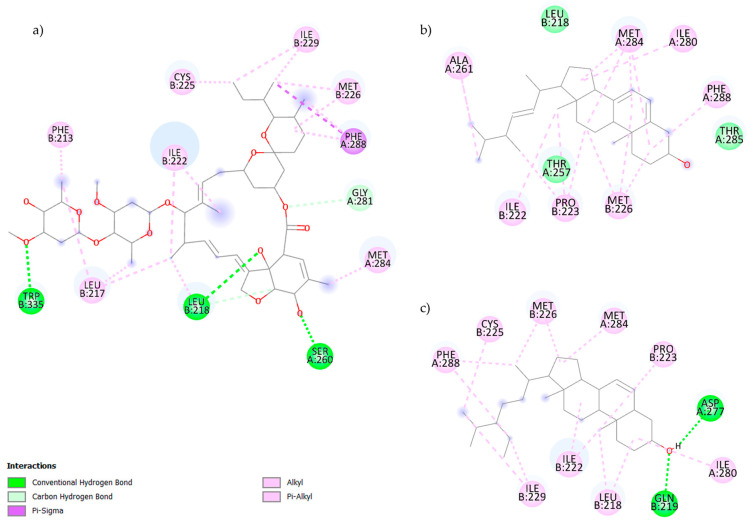
Molecular interactions of (**a**) ivermectin, (**b**) ergosterol, (**c**) β-sitosterol and the active site of the GluCl channel generated by molecular docking.

Figure 1 shows the interactions between the GluCl protein of *H. contortus* and the ligands ivermectin (a), ergosterol (b), and β-sitosterol (c), highlighting the participation of amino acids Thr257, Gln260, and Thr285 in chain A, and Val218 in chain B, recognized as essential residues of the active site [4]. In the case of ivermectin, several conventional hydrogen bonds are observed with residues Trp335, Leu218, Ser60, and Gly281, highlighting the ligand’s ability to form a robust polar anchor within the channel. Simultaneously, multiple interactions are formed with residues Phe213, Ile222, Ile229, Cys225, Met226, Leu217, Phe288, and Met284, as well as contacts with Val218, a key residue of the active site. This set of interactions is consistent with the compound’s amphiphilic nature and its well-established affinity for GluCl channels, where it acts as a highly effective allosteric modulator.

In panel b, corresponding to ergosterol, a binding mode dominated by hydrophobic interactions is identified, in which the steroidal nucleus is stabilized by alkyl and π-alkyl contacts with residues Ile222, Pro223, Met226, Met284, Ile280, Leu218, and Phe288, thereby generating a highly nonpolar environment consistent with its chemical structure. Although a single hydrogen bond is detected with Thr257, a crucial residue of the active site, the overall interaction is clearly determined by hydrophobic complementarity. This behavior is consistent with the predominantly nonpolar nature of sterols and their tendency to reside in low-polarity transmembrane cavities.

Finally, in the interaction of β-sitosterol with GluCl, the steroid’s hydroxyl group forms well-defined hydrogen bonds with Asp277 and Gln219, as well as an additional polar contact with Ile280, thereby generating specific recognition sites within the active site. Simultaneously, the rest of the molecule participates in multiple hydrophobic interactions with residues Met226, Met284, Ile222, Ile229, Leu218, Phe288, Cys225, and Pro223, including contacts with Val218 that help stabilize the steroid ring within the active site.

### 2.3. Molecular Dynamics Simulation

**Figure 2:**
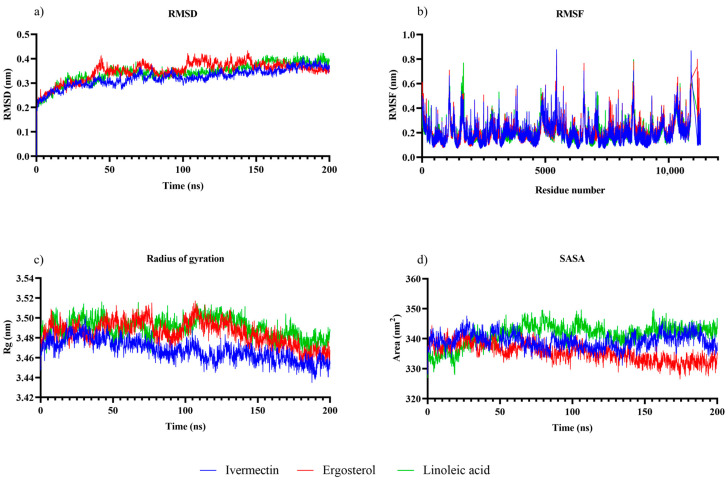
Structural stability and dynamic behavior of the protein–ligand complexes during 200 ns molecular dynamics simulations. Time-dependent profiles of (**a**) RMSD, (**b**) RMSF, (**c**) radius of gyration (Rg), and (**d**) solvent accessible surface area (SASA) for the complexes with ivermectin (blue), ergosterol (red), and linoleic acid (green).

Molecular dynamics (MD) simulations of 200 ns were performed for the ligands ivermectin, ergosterol, and linoleic acid, as these compounds showed the best effectiveness in the *in vitro* assays. Based on these simulations, the parameters of root mean square deviation (RMSD), root mean square fluctuation (RMSF), radius of gyration (Rg), and solvent-accessible surface area (SASA) were evaluated to characterize the temporal behavior and stability of the protein-ligand complexes (Figure 2).

Figure 2a shows that after 40 ns, the complexes reached stable trajectories and the RMSD values fluctuated between 0.30 and 0.40 nm during the 200 ns simulation. Of the three ligands, the ivermectin complex had the lowest and most constant RMSD values (~0.32 nm), suggesting a more rigid and stable binding mode. In contrast, the ergosterol complex showed slightly higher fluctuations, which may be associated with minor local rearrangements within the binding site.

The RMSF profiles (Figure 2b) revealed that most residues fluctuated between 0.10 and 0.30 nm, indicating limited flexibility in the protein backbone. In all three complexes, higher RMSF peaks (0.6–0.9 nm) were observed in specific regions, probably corresponding to loops or terminal ends, which are typically more flexible in nature. The similarity in RMSF patterns between the three complexes indicates that ligand binding did not alter the intrinsic dynamics of the protein. However, the ergosterol complex exhibited slightly greater fluctuations in certain regions, suggesting localized mobility near the binding site.

Regarding the radius of gyration (Rg), the values ranged from 3.46 to 3.52 nm for the three complexes (Figure 2c), remaining constant throughout the simulation. The ivermectin complex showed a slightly lower Rg value, suggesting a more compact protein conformation. The stability of Rg over time confirms that no global structural unfolding or expansion occurred, supporting the three complexes’ conformational equilibrium.

The SASA analysis (Figure 2d) showed small fluctuations within the 330–350 nm^2^ range, consistent with a stable solvent-exposed surface. The ergosterol complex showed slightly lower SASA values, suggesting more compact packing or partial burial of hydrophobic residues. In comparison, the linoleic acid complex showed slightly higher SASA values, indicating a configuration more exposed to the solvent. These differences, although subtle, may reflect variations in ligand-induced compactness at the binding site.

### 2.4. In Vitro Mortality Test

**Figure 3:**
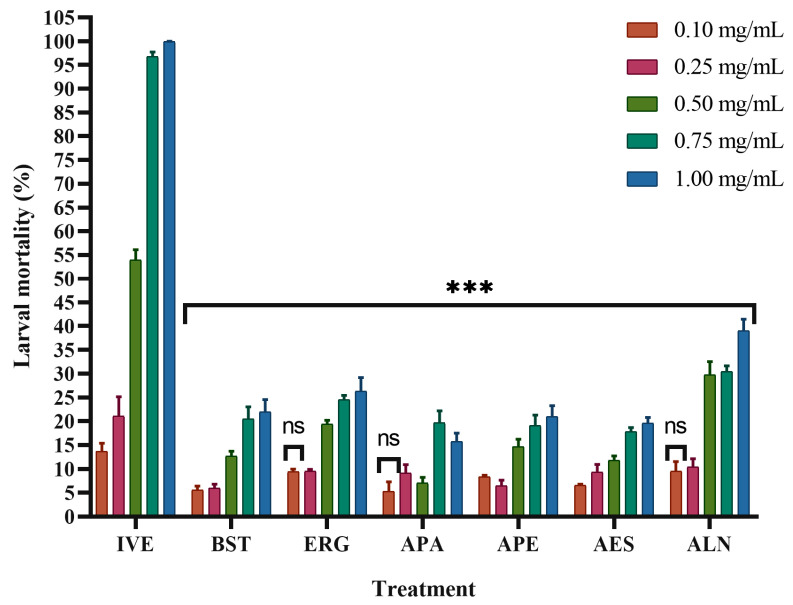
Effects of different treatments on the mortality of *Haemonchus contortus* infective L3 larvae. IVE (ivermectin), BST (β-sitosterol), ERG (ergosterol), APA (palmitic acid), APE (pentadecanoic acid), AES (stearic acid), and ALN (linoleic acid). Larvae were exposed to increasing concentrations (0.10–1.00 mg/mL), and mortality was assessed based on motility. Data are expressed as mean ± standard deviation (n = 4). Asterisks indicate significant differences compared with the DMSO-treated control group (one-way ANOVA followed by Tukey’s post hoc test): *** *p* < 0.001. “ns” indicates non-significant differences.

The results of the larval mortality test (Figure 3) show that ALN had the highest activity, with an effectiveness of 39% at a concentration of 1 mg/mL, followed by ERG (26%) and BST (22%) at the same concentration. None of these compounds reached 50% mortality within the concentration range evaluated (0.1–1.0 mg/mL). Consequently, their LC_50_ values were greater than the highest concentration tested, indicating lower larvicidal potency under the experimental conditions.

In contrast, ivermectin induced mortality levels exceeding 50%, allowing a direct determination of its LC_50_ value (0.43 mg/mL), which confirms its markedly higher toxicity compared to the tested fatty acids and sterols.

### 2.5. Larval Migration Test

**Figure 4:**
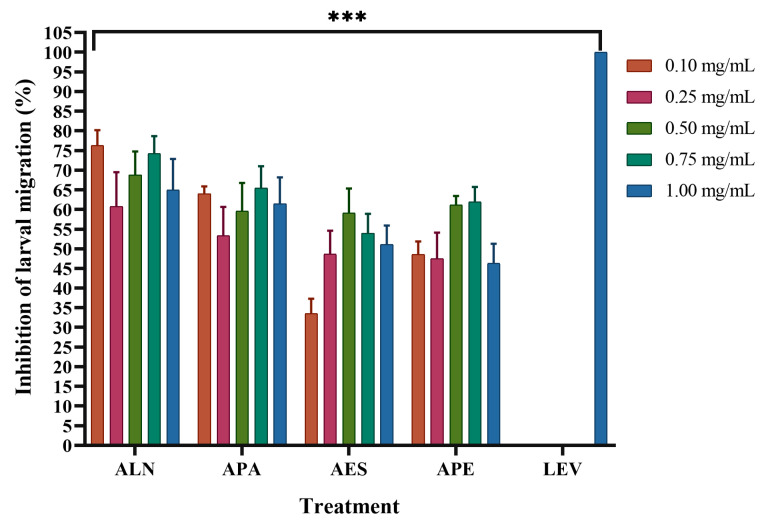
Effects of fatty acids on migration of *Haemonchus contortus* L3 infective larvae. LEV (Levamisole), APA (Palmitic acid), APE (Pentadecanoic acid), AES (Stearic acid), ALN (Linoleic acid). Data are expressed as mean ± standard deviation (n = 4). The asterisks indicate significant differences between treatments (ANOVA, Tukey’s post hoc test, *p* < 0.05): *** *p* < 0.001. Asterisks indicates significant differences between treated and control groups.

The larval migration test showed variable inhibition of *H. contortus* L3 motility depending on the compound and concentration tested (Figure 4). Across treatments, inhibition values ranged approximately between 20% and 75%. ALN exhibited the highest inhibition at the lowest concentration tested (0.10 mg/mL), whereas higher concentrations did not consistently increase the inhibitory effect. For the remaining compounds, migration inhibition values fluctuated across concentrations without a uniform increasing or decreasing pattern. Overall changes in larval migration did not follow a consistent dose-dependent trend for any of the evaluated treatments.

## 3. Discussion

In the present study, six commercial natural compounds (APA, APE, AES, ALN, BST, and ERG) were evaluated against infective L3 larvae of *H. contortus* resistant to benzimidazoles. These compounds were selected based on previous work by Pineda-Alegría et al. [9], who identified them in a bioactive methanolic fraction obtained from the edible mushroom *Pleurotus djamor* ECS-0123. In a subsequent study, Pineda-Alegría et al. [15] evaluated commercial APA, APE, AES, ALN, and BST—alone and in combination—against a susceptible strain of *H. contortus*, reporting 100% mortality at 10 mg/mL. Considering the growing problem of anthelmintic resistance, it is essential not only to explore alternative control strategies but also to clarify the biological relevance and possible mechanisms of action of such compounds, particularly when evaluated against resistant parasite strains.

The physicochemical and drug-likeness properties of the compounds were assessed using SwissADME. Although this tool primarily predicts mammalian pharmacokinetics and may not fully reflect the parasite context, it provides a useful preliminary evaluation of solubility, molecular flexibility, and chemical stability. These insights help prioritize compounds for further experimental evaluation and are consistent with approaches reported in previous studies on bioactive molecules, including antiparasitic compounds.

The glutamate-gated chloride channel (GluCl) is an invertebrate-specific ligand-gated ion channel and a validated pharmacological target of macrocyclic lactones such as ivermectin. Structurally, GluCl forms a pentameric transmembrane complex that regulates chloride influx upon glutamate binding, leading to neuronal hyperpolarization and inhibition of neuromuscular activity. In *H. contortus*, this channel plays a central role in locomotion and pharyngeal pumping, and its pharmacological modulation leads to flaccid paralysis and parasite death. Ivermectin binds to an allosteric site located between the M3 and M1 transmembrane helices, stabilizing the open conformation of the pore. Therefore, evaluating the interaction of sterols and fatty acids with GluCl is relevant from a mechanistic standpoint, as it allows the exploration of potential modulatory interactions with a well-established anthelmintic target [3,16,17].

It is important to note that each GluCl subunit contributes to ligand binding, channel gating, and structural stability. Since only two subunits are analyzed in this study, the cooperativity between subunits or possible binding sites present in other subunits could not be analyzed and their potential interactions could not be excluded. Therefore, the results of docking and molecular dynamics provide useful preliminary information but may not fully reflect ligand affinity or channel activation in vivo, highlighting the need for cautious interpretation in the context of resistant *H. contortus* larvae.

Docking analyses revealed that ERG and BST exhibited favorable binding energies and interacted with key residues such as Thr257, Gln260, Thr285, and Val218 within the GluCl channel [4]. Despite these favorable interactions, the limited *in vitro* effects observed indicate that binding affinity alone is insufficient to predict biological activity, underscoring the limitations inherent to this type of approach and strengthening the need for *in vitro* and *in vivo* studies, before only on docking analysis as they rely on static protein conformations and simplified energy estimations. Experimental factors such as compound solubility, aggregation, stability, and limited penetration through the larval cuticle may reduce the effective concentration at the target site.

Given the lipophilic nature of sterols, limited penetration through the nematode cuticle may further compromise their ability to exert a direct inhibitory effect on GluCl *in vivo*. Although several studies have reported antiparasitic properties of BST and ERG against different parasite species, including *H. contortus* [13,15], differences in nematode species, developmental stage, compound formulation, and experimental design limit direct comparisons with the present study. Sterols have been reported to exert indirect antiparasitic effects through alterations in membrane structure and changes in immunomodulatory effects in the host [18,19,20], mechanisms that cannot be captured in *in vitro* larval mortality assays.

The development of new anthelmintic candidates can significantly benefit from rational design strategies based on molecular structure and mechanism of action. Such approaches are applicable throughout the discovery process, from the identification of bioactive compounds in natural extracts to their structural optimization for improved biological performance [21]. In this context, the integration of molecular docking and molecular dynamics simulations used in the present study contributes to a mechanistic framework that helps interpret *in vitro* observations beyond simple activity screening.

Advances in genomic and structural biology technologies have further enhanced the identification of drugs targeting specific molecular sites through bioinformatics tools, facilitating the integration of biological knowledge with therapeutic development [22]. The use of GluCl as a validated molecular target, combined with computational analyses, aligns with these strategies and supports a rational interpretation of the differential biological effects observed among fatty acids and sterols.

The integration of molecular dynamics (MD) simulations provided additional mechanistic insight into ligand–protein interactions beyond static docking predictions. All ligand-GluCl complexes remained structurally stable throughout the simulations, with ivermectin showing the most compact and rigid interaction pattern, consistent with its established role in stabilizing the open pore conformation of GluCl channels [23]. ERG and ALN also maintained stable associations, although with greater local flexibility. These observations indicate that stable binding and limited conformational fluctuations of the receptor are not sufficient to ensure strong biological efficacy, as none of the compounds studied was able to influence the migration of resistant *H. contortus* larvae, confirming the need to conduct *in vitro* and *in vivo* studies before any possible recommendation can be made for their use in productive settings.

Previous studies have reported nematicidal activity for several fatty acids evaluated here. Stadler et al. [24] described significant nematicidal effects of palmitic, stearic, and linoleic acids against *Caenorhabditis elegans*, with LD_90_ values ranging from 50 to 100 µg/mL. Similarly, Zhang et al. [8] reported inhibitory effects of fatty acids on egg hatching and juvenile mortality in *Meloidogyne incognita*, while Panda et al. [10] identified ALN as the main nematicidal component in extracts of *Holigarna caustica*, with an IC_50_ of 0.2 µg/mL against *C. elegans*. These findings contrast with the results of the present study, where none of the compounds and doses studied were able to exert nematocidal effects and this can be explained by the resistant nature of the *H. contortus* strain used in this study, indicating the importance of understanding the nematode biology in the design of future treatments.

In the larval migration assay, fatty acids exhibited a predominantly nematostatic effect by reducing larval motility, although a clear dose-response relationship was not observed across the concentration tested. This response pattern is not consistent with a classical receptor-mediated mechanism and suggests that the inhibition of larval migration may arise from non-specific physicochemical effects rather than direct modulation of the glutamate-gated chloride channel (GluCl). Proposed modes of action include disruption of the nematode’s hypodermis cuticle through detergent-like activity or interactions with lipophilic regions of plasma membranes.

In addition, physicochemical stress related to compound aggregation or limited solubility at higher concentrations may further contribute to the lack of a monotonic dose-dependent response. Although ALN has been reported to modulate ionotropic neurotransmitter receptors, including NMDA and GABA receptors [25,26], the overall findings indicate that fatty acids likely exert multifactorial and indirect effects rather than acting as potent single-target anthelmintic agents.

The *H. contortus* strain used in this study is resistant to benzimidazoles and may harbor specific genetic adaptations that influence susceptibility to alternative compounds. Anthelmintic resistance varies among parasite populations and is shaped by treatment history, genetic diversity, and environmental pressures. These factors likely contribute to the reduced efficacy observed compared with studies performed on susceptible strains.

Overall, this study confirms the importance of integrating larval assays with molecular docking and molecular dynamics simulations to gain deeper insight into the biological effects of selected fatty acids and sterols. This combined *in vitro* and *in silico* approach facilitates a more accurate interpretation of antiparasitic activity and underscores the need for caution when relying solely on computational affinity predictions, particularly in drug-resistant parasite models.

## 4. Materials and Methods

### 4.1. ADMET Analysis

The SwissADME web server (Swiss Institute of Bioinformatics, Lausanne, Switzerland; https://www.swissadme.ch/, accessed on 11 March 2024) was used to evaluate the pharmacokinetic profile and potential metabolism of the selected compounds. To check the properties of the compounds, the simplified molecular input format (SMILES) and the structural data file (SDF) were used as input.

### 4.2. In Silico Analysis

#### 4.2.1. Obtaining Molecular Structures

**Figure 5:**
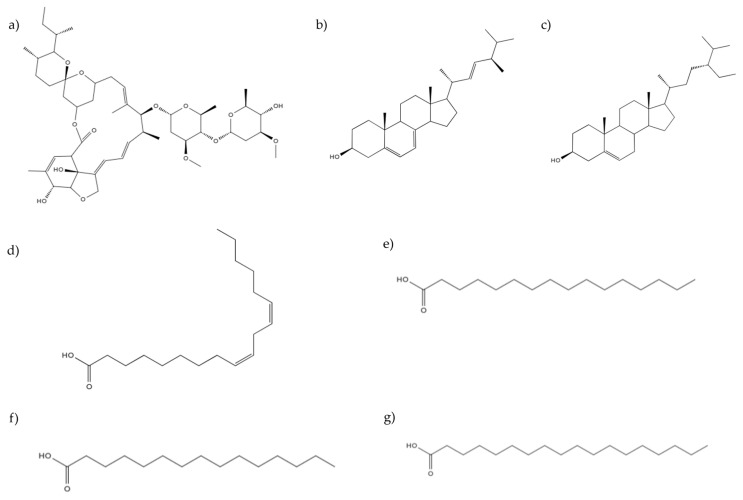
Chemical structures of the compounds ivermectin (**a**), ergosterol (**b**), β-sitosterol (**c**), linoleic acid (**d**), palmitic acid (**e**), pentadecanoic acid (**f**), and stearic acid (**g**) evaluated in this study obtained from the PubChem database.

The chemical structures of the ten compounds evaluated in this study, including the reference standards, were obtained from the PubChem database (https://pubchem.ncbi.nlm.nih.gov, accessed on 18 March 2024) and are shown in Figure 5. All structures were downloaded in SDF format and subsequently used for structural optimization and *in silico* analyses.

#### 4.2.2. Structural Optimization with Avogadro

The downloaded ligands were imported into Avogadro software version 1.2.0 (Avogadro Project, Pittsburgh, PA, USA) for review and optimization. First, the correct assignment of bonds and protonation states was verified. Next, molecular energy minimization was performed using the MMFF94 force field, enabling a more stable, lower-energy structural conformation to be obtained for molecular coupling analysis. In addition, any possible irregularities in molecular geometry were corrected, and the correct atomic charges were ensured.

#### 4.2.3. Preparation of *H. contortus* Proteins

The primary structure of the glutamate-gated chloride channel (GluCl) of *H. contortus* (UniProt ID: P91730) was selected and sent to the BLASTP server (National Center for Biotechnology Information, Bethesda, MD, USA; https://blast.ncbi.nlm.nih.gov/, accessed on 26 March 2024) to search the Protein Data Bank (PDB) database for the crystallographic structure with the highest sequence identity and coverage. The GluCl channel structure co-crystallized with ivermectin from the nematode *Caenorhabditis elegans* (PDB ID: 3RHW) [17] was selected, showing 56.83% amino acid identity. Chains A and B, located in the transmembrane domain, were prepared for molecular docking studies using the PyMol version 3.0.5 (Schrödinger, LLC, New York, NY, USA) tool. Solvent molecules and co-crystallized ligands were removed, and hydrogen atoms were added to the protein model.

#### 4.2.4. Molecular Docking

For molecular docking analysis, the search box was first set up in AutoDockTools version 1.5.7 (The Scripps Research Institute, La Jolla, CA, USA) to ensure the volume covered the protein’s active site. The x, y, and z coordinates were set, along with a size of 60 × 60 × 60, centered on residues T257, Q260, and T285 located in chain A, in addition to residue V218 in chain B, to capture possible interactions between the ligand and the protein. The docking parameter file (dpf) was generated with specific settings for the molecular docking run. AutoGrid version 4.2.6 (The Scripps Research Institute, La Jolla, CA, USA)was used to create energy maps of the receptor protein’s atoms within the defined search box. Subsequently, molecular docking was performed using AutoDock4 version 4.2.6, with 100 independent runs to obtain multiple ligand conformations within the active site. The best binding mode for each molecule was selected based on the lowest free binding energy and the largest cluster size. Finally, the docked complexes were examined in PyMOL version 3.0.5 and Discovery Studio version 24.1.0.23298 (BIOVIA, Dassault Systèmes, San Diego, CA, USA) to detect non-bonded interactions.

#### 4.2.5. Molecular Dynamics Simulation

After identifying the most favorable docking pose for each target protein, molecular dynamics (MD) simulations were conducted to evaluate the conformational stability and intermolecular interactions of the resulting complexes. System preparation was performed using the CHARMM-GUI server ((version 1.9; Harvard University, Cambridge, MA, USA; https://www.charmm-gui.org/, accessed on 15 July 2025) under physiological conditions (pH 7.4, 310.15 K), employing the TIP3P water model and 0.15 M NaCl to match the physiological ionic strength. Each complex was centered within a cubic box, with at least 10 Å between the protein surface and the box edges and was parameterized using the CHARMM36m force field. Input topology and coordinate files were generated for GROMACS 2019.6 [27,28,29].

Energy minimization was performed using the steepest descent algorithm until the maximum force (Fmax) was below 1000 kJ/mol nm. Equilibration was carried out in two stages: first under NVT conditions (constant number of particles, volume, and temperature), followed by NPT conditions (constant number of particles, pressure, and temperature). Temperature coupling was maintained with the Vrescale thermostat, and pressure stabilization with the Parrinello—Rahman barostat. Production simulations were conducted under the NPT ensemble, with three independent 200-ns replicates executed in GROMACS 2019.6. All simulations were run on the Xiuhcóatl supercomputing cluster at the National Laboratory of High-Performance Supercomputing (LANCAD) [30,31,32,33].

### 4.3. In Vitro Assays

#### 4.3.1. Commercial Compounds

The compounds evaluated: pentadecanoic acid (CAS 1002-84-2), stearic acid (CAS 57-11-4), palmitic acid (CAS 57-10-3), linoleic acid (CAS 60-33-3), β-sitosterol (CAS 83-46-5), and ergosterol (CAS 57-87-4) were purchased from SIGMA-ALDRICH^®^ (Toluca de Lerdo, State of Mexico, Mexico).

#### 4.3.2. *Haemonchus contortus* Donor Animal

The nematode *H. contortus* L3 was produced in a 3-month-old Pelibuey ram, free of parasites, at the FMVZ-UADY nematode production facilities. The donor was artificially infected with 350 infective L3 larvae per kilogram of body weight of the benzimidazole-resistant Paraíso strain, originally obtained from a sheep farm in Yucatán, Mexico, as described in Chan-Pérez et al. [34]. The donor was housed in a metabolic cage located inside a concrete-floored pen before and during L3 production. The donor lamb was offered a balanced daily diet in its metabolic cage and ad libitum access to drinking water. The animals were only handled by authorized personnel from the production facility. Twenty-one days after infection, fecal samples were collected directly from the animals using the McMaster technique to confirm patent infection [35].

#### 4.3.3. Obtaining Infective Larvae Without Sheaths

To obtain infective larvae, feces were obtained from the fecal collection device of the metabolic cage of the donor animal using a plastic container. The feces were transferred to coprocultures, which were incubated at 27 ± 1 °C for 5 days. After the incubation period, the larvae were recovered using the Baerman funnel technique [34]. Once the L3 stage was reached, the larvae were exposed to a 0.187% sodium hypochlorite solution for 5 min to remove the sheaths. Subsequently, three washes with distilled water were performed for 1.5 min at 3500 rpm to remove the sodium hypochlorite [36]. The L3 larvae were stored in ventilated cell culture flasks at 8 ± 2 °C until use.

#### 4.3.4. Larval Mortality Test

Solutions of each compound were prepared in different concentrations (0.10, 0.25, 0.50, 0.75, and 1.00 mg/mL) using 0.05% dimethyl sulfoxide (DMSO; Sigma-Aldrich, St. Louis, MO, USA) as a diluent.

To evaluate larval mortality, 96-well microtiter plates were used, each well containing a mixture of 50 µL of the corresponding treatment and 50 µL of an aqueous suspension containing 100 L3 larvae. Ivermectin (IVERPLUS 1% injectable solution, Vet’s Pharma, Mexico City, Mexico) was used as a positive control at the same concentrations as the compounds, and 0.05% DMSO was used as a negative control. Four replicates were performed for each treatment, and the plates were then covered with aluminum foil and incubated at 28 ± 1 °C for 72 h. At the end of the confrontation time, 10 aliquots of 10 μL were taken from each well and placed on a slide, which was observed with an optical microscope (40× and 10×) to quantify the number of live and dead larvae. The percentage of larval mortality at each concentration was calculated using the following formula [36]:

$$Larvae\;mortality\;\%=\frac{Number\;of\;dead\;larvae}{Number\;of\;live\;larvae+Number\;of\;dead\;larvae}\times 100$$

#### 4.3.5. Larval Migration Test

The larval migration test was performed only with four fatty acids: palmitic, pentadecanoic, stearic, and linoleic. The technique was adapted from Demeler et al. [37]. Twenty-four-well microtiter plates were used; each well contained 200 μL of each compound at concentrations of 0.10, 0.25, 0.50, 0.75, and 1.00 mg/mL and 200 μL of a suspension of L3 larvae without sheaths. Levamisole (Vermidazole-15, Prossiter S.A de C.V, Atizapán de Zaragoza, México) at 1.00 mg/mL was included as a positive control because it inhibits larval motility. The plates were incubated at 28 °C for 72 h. After the incubation period, the contents of each well were filtered through sieves (25 μm mesh) into new plates called migration plates. The migration plates were incubated at 28 °C for 24 h to allow the larvae to migrate through the sieve. The sieves were then removed from the wells, and an optical microscope (40× and 10×) was used to count the larvae that had passed through the sieve (migrated) and those that remained trapped in the sieve (non-migrated). The larval migration percentage (LM%) for each concentration was calculated using the following formula [37]:

$$LM\%=\frac{Non-migrated\;Larvae}{Migrated\;larvae+Non-migrated\;larvae}\times 100$$

#### 4.3.6. Data Analysis

The results of both assessments (mortality and migration) were analyzed using a normality test and transformed using the arcsine square root. An analysis of variance (ANOVA) was performed, followed by a Tukey’s mean comparison test (*p* < 0.05) in GraphPad Prism 8 software.

## 5. Conclusions

Molecular docking and molecular dynamics analyses indicated that sterols such as ergosterol and β-sitosterol can interact stably with the glutamate-gated chloride channel (GluCl) of *H.contortus,* although these interactions did not translate into strong biological efficacy *in vitro*. Fatty acids, particularly linoleic acid, exhibited nematostatic effects by reducing larval motility, suggesting indirect or physicochemical modes of action rather than classical receptor-mediated mechanisms.

Taken together, the results highlight the complexity of linking *in silico* affinity predictions with biological outcomes, especially when evaluating compounds against drug-resistant parasite strains. This study demonstrates the value of integrating *in vitro* assays with computational approaches to provide mechanistic insights while also emphasizing the limitations of relying solely on binding stability as a predictor of anthelmintic activity. Further complementary studies, including refined *in silico* analyses and *in vivo* evaluations, are required to better assess the biological relevance of these metabolites.

## Figures and Tables

**Table 1:** Analysis of the pharmacokinetic properties of the compounds evaluated.

Compound	Molecular Weight (g/mol)	Rotatable Links	H Acceptors	H Donors	Bioavailability	Synthetic Accessibility
APA	256.42	14	2	1	0.85	2.31
APE	242.40	13	2	1	0.85	2.20
AES	284.48	16	2	1	0.85	2.54
ALN	280.45	14	2	1	0.85	2.54
BST	414.71	6	1	1	0.55	6.30
ERG	396.65	4	1	1	0.55	6.58

**Table 2:** Binding energy values of the compounds evaluated in the GluCl channel of *H. contortus*.

Compound	Binding Energy (kcal/mol)
Ivermectin (IVE)	−13.57
Ergosterol (ERG)	−10.25
β-sitosterol (BST)	−10.18
Linoleic acid (ALN)	−4.59
Palmitic acid (APA)	−4.47
Pentadecanoic acid (APE)	−4.36
Stearic acid (AES)	−4.22

## Data Availability

The original contributions presented in this study are included in the article. Further inquiries can be directed to the corresponding author.
